# Enhanced Sorption for the Oil Spills by SDS-Modified Rice Straw

**DOI:** 10.3390/gels9040285

**Published:** 2023-04-01

**Authors:** Yongfei Li, Jiangbo Liu, Wenjuan Li, Miao Dou, Liwa Ma, Qian Wang, Bin Zhao, Gang Chen

**Affiliations:** 1 State Key Laboratory of Petroleum Pollution Control, Xi’an Shiyou University, Xi’an 710065, China; 2 Shaanxi Province Key Laboratory of Environmental Pollution Control and Reservoir Protection Technology of Oilfields, Xi’an Shiyou University, Xi’an 710065, China; 3 Oil & Gas Technology Research Institute Changqing Oilfield Company, Xi’an 710060, China; 4 Xi’an ChangQing Petrochemical Corporation Co., Ltd., Xi’an 710018, China; 5 Department of Statistics, North Dakota State University, Fargo, ND 58102, USA

**Keywords:** rice straw, acid treatment, surface modification, oil absorption

## Abstract

Frequent oil spills have caused serious consequences to the ecosystem and environment. Therefore, in order to reduce and eliminate the impact of oil spills on biology and the environment, oil spill remediation materials must be considered. As a kind of cheap and biodegradable natural organic cellulose oil-absorbing material, straw has an important practical significance in the treatment of oil spills. In order to improve the ability of rice straw to absorb crude oil, rice straw was first treated with acid and was then modified with sodium dodecyl sulfate (SDS) through a simple charge effect. Finally, the performance of oil absorption was tested and evaluated. The results illustrate that the oil absorption performance was greatly improved under the conditions of 10% H_2_SO_4_, for a 90 min reaction at 90 °C, under 2% SDS, and reacted for 120 min at 20 °C, and the rate of adsorption for rice straw to crude oil was raised by 3.33 g/g (0.83 to 4.16). Then, the rice stalks before and after the modification were characterized. Contact angle analysis shows that the modified rice stalks display better hydrophobic–lipophilic properties than unmodified rice stalks. The rice straw was characterized by XRD and TGA, and the surface structure of the rice straw was characterized by FTIR and SEM, which explain the mechanism of surface-modified rice straws with SDS to improve their oil absorption capacity.

## 1. Introduction

Currently, the frequency of marine oil spills is increasing under the increasing frequency of marine transportation and oil and gas extraction activities [1,2]. There are several typical events such as the Gulf of Mexico [3], the Saronic Gulf incident [4], the North American Atlantic [5], and the Santorini [6]. These oil spills have caused serious consequences to our marine ecosystem and ecological environment. Therefore, oil spill remediation materials and technologies have been attracting much attention so as to reduce and eliminate the influence of oil spills on our biology and environment. Nowadays, the materials and technologies that are commonly used in oil spill remediation are mainly divided into four different types: (1) chemical methods (dispersants and curing agents), (2) in situ combustion, (3) bioremediation, and (4) mechanical recycling (oil boom, oil skimming boom, and adsorbent) [7,8]. Among them, adsorbent materials are more attractive for oil spill cleaning because they can collect and completely remove the oil from the surface of the water without adversely affecting the environment.

There are a few hydrophobic and lipophilic adsorbents [9,10,11,12,13,14], including magnetic particles [15,16,17], textile [18,19,20,21], and modified commercial sponges [22,23,24], which have been applied for oil adsorption. At present, materials with high oil absorption are generally industrially synthesized as high oil-absorbing resin materials [25]. However, such materials not only have high production costs and cannot be used for secondary use and biodegradation, but they are likely to cause environmental pollution. However, rice straw, as a cheap and biodegradable natural organic cellulose oil-absorbing material, is an abundant cellulosic product from the production of farming in many countries, and it has great practical significance to be prepared as an oil spill absorbent [17,26]. It is not only a valid approach to take full advantage of our resources, but it is also a good way to settle pollution issues from the sources. Nevertheless, the absorption ability of oil and the hydrophobic properties of organic plant fibers are poor. Thus, chemical modification methods are indispensable for enhancing their adsorption capacity for crude oil [27,28,29].

Therefore, in this paper, the crop waste of rice straw with unique advantages of being low in price, non-toxic, harmless, and non-polluting was used to solve the above problems. Our objective in this study was to increase the oil adsorption capacity and acquire a highly efficient and modified straw oil-absorbing material via a simple, easy-to-operate, and low-investment method. However, the oil absorption rate of natural absorbent material rice straw is not ideal. Thus, in order to improve the oil absorption rate and obtain a modified straw oil-absorbing material with high efficiency, the rice straw was physically processed with cutting into pellets pretreatment and was modified by surface modification with composite ion surfactant treatment after wetting with acid.

## 2. Results and Discussion

### 2.1. Mechanism of Composite Surface Modification

**Figure 1:**
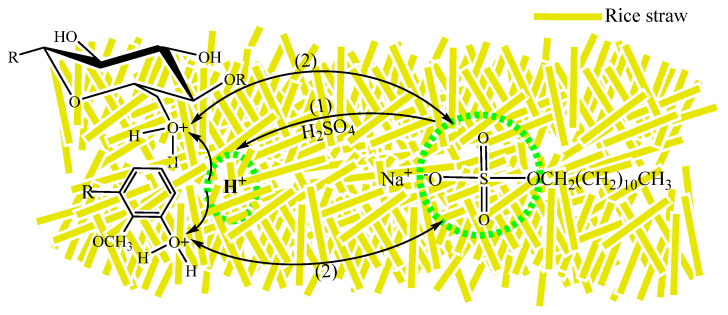
Mechanism of composite surface modification.

The schematic diagram of modification is showed in Figure 1. Firstly, the straw was pretreated with acid, and the acid cellulose and lignin formed by cellulose and lignin on the surface of the rice straws reacted with acid, which made the cellulose and lignin have strong electron withdrawing properties and produce unstable cations on the surface of the fiber [30,31].

For the modification of anionic surfactants, the reaction focuses on the adsorption of anion and cation. The raw materials should be washed to neutrality with pure water after the acid treatment, because the surfactant easily loses activity and precipitates out in the acid medium. Then, the negatively charged part of the anionic surfactant binds to the surface of the positively charged cellulose and lignin to increase the oil absorption after the anionic surfactant.

### 2.2. Influence of Modification Conditions on Oil Absorption

#### 2.2.1. Effect of Acid Modification Conditions

**Figure 2:**
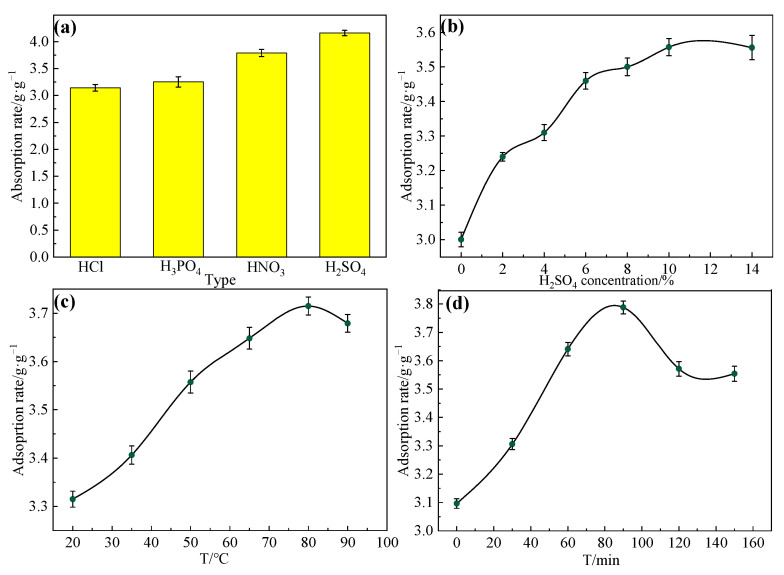
(**a**) Effect of acid type under the concentration of 10%, and reacting for 90 min at 80 °C. (**b**) Effect of H_2_SO_4_ concentration under reacting for 90 min at 80 °C. (**c**) Effect of acid treatment temperature under the concentration of 10% H_2_SO_4_ and reacting for 90 min. (**d**) Effect of acid treatment time under the concentration of 10% at 80 °C.

Firstly, the influence of different acids was explored under the concentration of 10%, reacting for 90 min, at 80 °C. The effect of the acid type was examined. Figure 2a exhibits that the effect on the rice straw was different because of different levels of acidity. Among them, H_2_SO_4_ had the strongest acidity and the highest oil absorption rate, and the oil absorption rate reached 4.16 g/g. Therefore, H_2_SO_4_ was the optimal choice for acid treatment.

Then, the influence of H_2_SO_4_ was investigated at the concentration of 0 to 14% after reacting for 90 min at 80 °C. The result indicated that the oil absorption rate increased as the concentration of H_2_SO_4_ increased, as can be seen in Figure 2b. The oil absorption rate is the highest when the concentration of H_2_SO_4_ reaches 10%. The overall oil absorption rate increases from 3.00 g/g to 3.56 g/g as the H_2_SO_4_ concentration increases from 0 to 10%. It is ascribed that the reaction will be easy to carry out when the more cations are produced by reacting with the cellulose and lignin on the surface of the rice straw as the H_2_SO_4_ concentration increases. Therefore, 10% H_2_SO_4_ was optimal.

In Figure 2c, the influence of the temperature for alkali treatment on the oil adsorption ability was investigated with 10% H_2_SO_4_, and it reacted for 90 min. The effect of the acid treatment temperature on the oil absorption ability was investigated. Figure 2 highlights how the oil absorption rate increased from 3.40 to 3.79 g/g when the temperature for acid treatment was <80 °C. However, the oil absorption rate did not increase obviously, and it tends to be stable when it is >80 °C. This is because the oil absorption has reached saturation when the temperature is at 80 °C. Therefore, 80 °C was the optimal acid treatment temperature.

To optimize the H_2_SO_4_ reaction time, the influence of different timings was investigated under the condition of 10% at 80 °C. Figure 2d demonstrates that the oil absorption rate increased from 3.10 g/g to 3.78 g/g as the duration of the alkali treatment changed from 0 to 90 min. Moreover, the oil absorption rate decreased significantly from 3.78 g/g to 3.55 g/g when the time was >90 min. This is attributed to the rapid adsorption equilibrium, and so, the oil absorption rate continued to increase when the time changes from 0 to 90 min, whereas the adsorption balance will break and the absorption rate will reduce when the time is >90 min. Therefore, 90 min was selected as the length of acid treatment.

#### 2.2.2. Effect of Anionic Surfactant Modification Conditions

**Figure 3:**
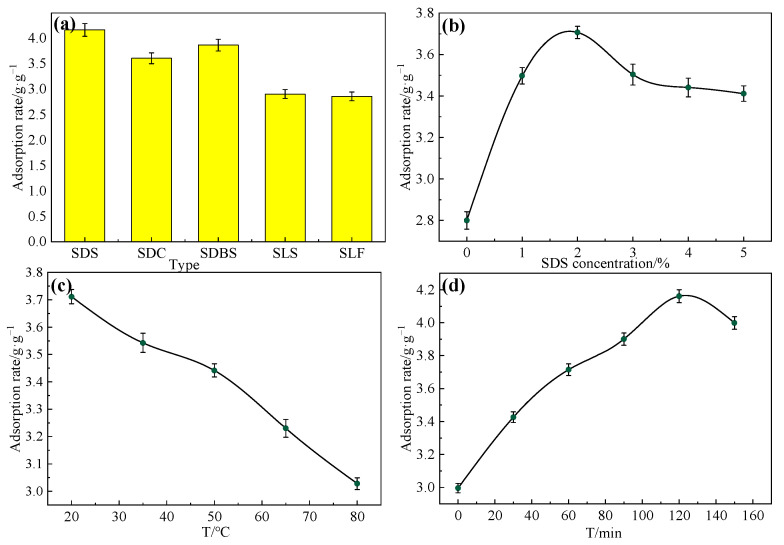
(**a**) Effect of anionic surfactant type under the concentration of 2% and reacting for 120 min at 20 °C. (**b**) Effect of SDS concentration under 2 °C and reacting for 120 min. (**c**) Effect of surface modification temperature under 2% CTAC and reacting for 120 min. (**d**) Effect of surface modification time under the concentration of 2% at 20 °C.

In order to determine an anionic surfactant with the highest oil absorption efficiency, we studied the influence of the quaternary ammonium cationic surfactant on the oil adsorption at a concentration of 2% and reacting for 120 min at 20 °C. The effect of the anionic surfactant type was also investigated. As shown in Figure 3, the influence of the different anionic surfactants on rice straw was different, and the oil absorption rate of the surface modification with sodium dodecyl sulfate (SDS) was higher than the others. The oil absorption rate of SDS was 4.16 g/g, for sodium dodecyl carboxylate (SDC) it was 3.60 g/g, for sodium dodecylbenzene sulfonate (SDBS) it was 3.86 g/g, for sodium Lauryl stearate (SLS) it was 2.90 g/g, and for sodium lauryl fat (SLF) it was 2.85 g/g.

The concentration of SDS was investigated at 20 °C and reacting for 120 min by varying the concentration from 1% to 5%. The effect of SDS was examined by changing the concentration (1–5%). The oil absorption rate increased rapidly from 2.80 g/g to the highest value of 3.71 g/g when the concentration of the SDS increased from 1% to 2% (Figure 3b). The main reason for this is attributed to the fact that the oil absorption reached saturation when the concentration was 2%. However, the resolution emerged when the concentration continued to increase, which greatly reduced the oil absorption rate. Thus, a concentration of 2% is optimal for SDS.

Then, the modification temperature of SDS was investigated at 2% CTAC (cetyltrimethylammonium chloride) and reacting for 120 min. As shown in Figure 3c, the highest oil absorption rate can be reached at 3.71 g/g when the temperature is 20 °C. The oil absorption rate reduces from 3.71 g/g to 3.03 g/g as the temperature rises. This is because that anionic surfactants tend to cause inactivation at high temperatures, and so the reaction is prone to be carried out at low temperatures. Therefore, 20 °C was the optimal temperature for surface modification.

Finally, the influence modification time for SDS was studied under the conditions of 2% and at 20 °C. Figure 3d illustrates that the absorption rate obviously increased (3.00 g/g to 4.16 g/g) as the surface modification time varied from 0 to 120 min, whereas the oil absorption rate decreased from 4.16 g/g to 4.00 g/g when the time exceeded 120 min. This is due to the ion movement of the anionic surfactant being the most violent after the reaction of 120 min, and the crude oil will be resolved. Thus, 120 min was the optimal time for surface modification.

#### 2.2.3. Characterization of Rice Straw

**Figure 4:**
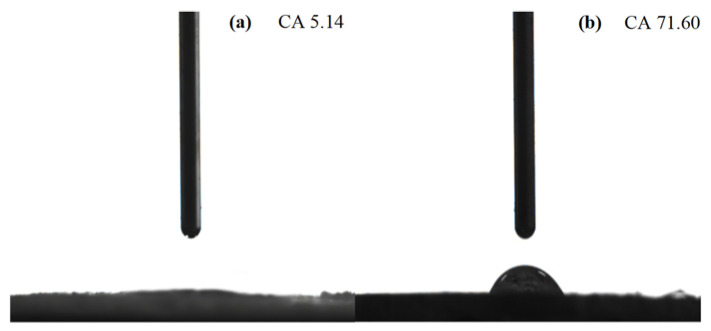
Contact angle of rice straw: (**a**) unmodified, (**b**) modified with SDS under the concentration of 2%, and reacting for 120 min at 20 °C.

Figure 4 exhibits the contact angle between the modified and unmodified rice straws with pure water. Figure 4a illustrates the high hydrophilicity of the unmodified rice straw. Figure 4b indicates that the high hydrophobicity but low hydrophilicity of the rice straw after modification and the contact angle with pure water increased from 5.14° to 71.60°, which demonstrates that the modified rice straw has hydrophobic characteristic [32,33,34].

**Figure 5:**
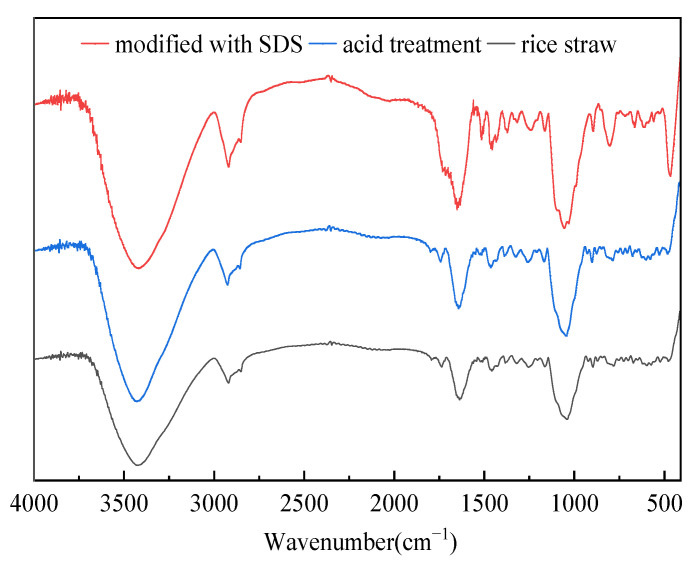
FT-IR spectra of rice straw, acid treatment, and modified with SDS.

Then, the unmodified and modified rice straws were characterized using an FT-IR spectrometer. As shown in Figure 5, no obviously different peaks existed between the unmodified and modified rice straw. However, the intensity of the C–H band at 2800–2900 cm^−1^ increased [35]. This is because of the charge effect of the cationic lignin and cellulose on the surface of the rice stalks and anionic surfactants under the action of sulfuric acid.

**Figure 6:**
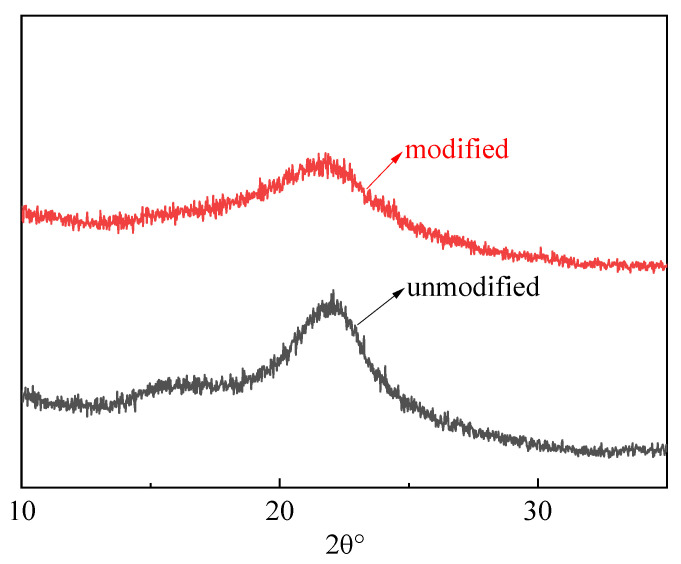
XRD pattern of rice straw, unmodified and modified.

The XRD patterns of unmodified and modified rice straw in Figure 6 exhibit cellulose Iβ characteristic peaks at around 2θ of 21.5°, representing the d 200 crystallographic planes of the monoclinic lattice structure [36,37]. Moreover, the crystallinity after treatment is weaker than before the treatment, which is due to the intermolecular and intramolecular bonds of cellulose that were destroyed by the modification, leading to decreases in the crystallinity [38].

**Figure 7:**
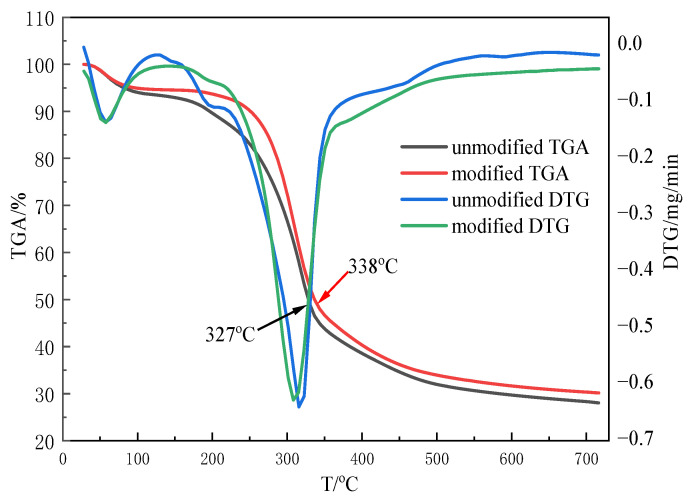
Thermograms of unmodified and modified.

In order to determine their thermal natures, the unmodified rice straw and modified rice straw were subjected to derivative TGA in an N_2_ atmosphere. Figure 7 illustrates the thermograms of native rice straw and modified straw samples. The TGA curves demonstrate an obvious decrease from 30 °C to 100 °C due to a loss of moisture. After that, the unmodified straw starts to decompose at 181 °C; however, the modified straw sample starts to decompose at 238 °C. At 50% weight loss, the decomposition temperature occurs at 327 °C for the nature rice straw and at 338 °C for the modified straw sample. This increasing trend of the decomposition temperature illustrates that the thermal stability of the modified rice straw is higher than that of the unmodified rice straw [39].

**Figure 8:**
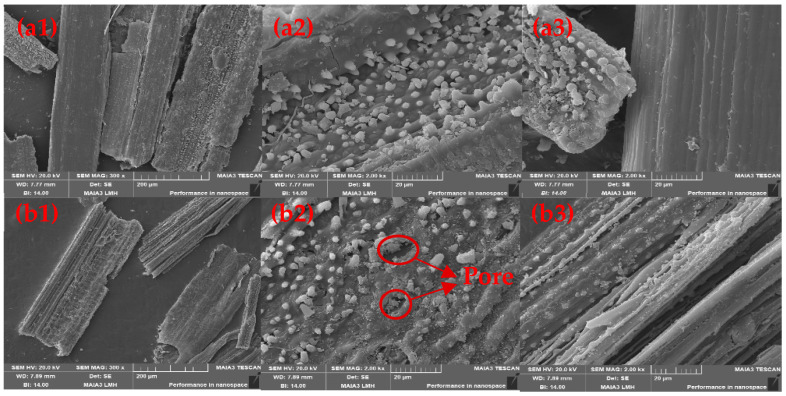
SEM images: (**a1**–**a3**) unmodified, (**b1**–**b3**) modified with SDS under the concentration of 2%, and reacting for 120 min at 20 °C.

Finally, to investigate the morphology and microstructure of rice straw before and after modification, they were characterized with SEM. Figure 8 exhibited the tubular structures of unmodified (a1) and modified (b1) rice straw. Figure 8b2,b3 obviously shows the porous and loose web structure of the modified rice surface fibers. By comparing Figure 8a3,b3, we can conclude that the surface area of the rice straw increased, which enhanced the hydrophobic properties [40] and supports previous contact angle measurement.

## 3. Conclusions

The adsorption capacity of crude oil was evaluated through specific oil adsorption experiments. The optimized and determined preparation conditions were as follows: 10% H_2_SO_4_ reacted for 90 min at 80 °C, and 2% SDS reacted for 120 min at 20 °C, and the adsorption rate of the rice straw of crude oil increased from 0.83 g/g to 4.16 g/g. The reason for the increased crude oil sorption ability of the modified material was explained by the theoretical level of the size of the contact angle and the principle of wettability. The characterization methods of XRD, FTIR, SEM, contact angle measurement, and thermogravimetric analysis (TGA) further proved that the modified rice straw had hydrophobic characteristics. This study uses modified straw as a cheap and biodegradable natural organic cellulose oil-absorbing material. It is not only an effective way to make full use of resources, but it is also an effective measure to solve the pollution problem from the source. However, its application scenario needs to be further expanded.

## 4. Materials and Methods

### 4.1. Materials

The oil was supplied by Chang 2 reservoir of Yanchang Oilfield, and properties of which are shown in Table 1. SDS, SDC, SDBS, SLS, SLF, and other reagents were purchased with analytical grade. Rice straw was collected from farmland around Jingmen City, Hubei Province.

### 4.2. Preparation of Rice Straw

#### 4.2.1. Pretreatment of Rice Straw

**Figure 9:**
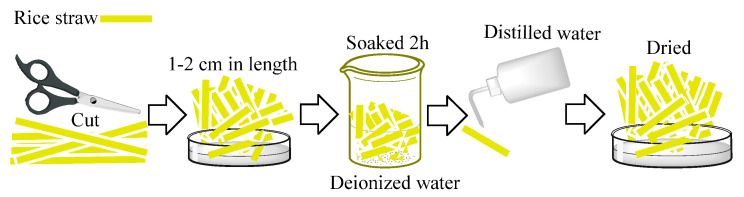
Preparation diagram of rice straw.

The rice stalks were pretreated before modification, the pretreatment process was shown in Figure 9, and the specific treatments are as follows: the rice straw was cut into pieces 1–2 cm, and they were soaked in deionized water for 2 h, then they were washed once by distilled water and following this, they were placed into an oven at 65 °C at a constant weight [41].

#### 4.2.2. Modification of Rice Straw

Firstly, the rice straw was treated by acid. A total of 50 mL acid was poured into the beaker with oven-dried straw, then beaker was put in the water bath at 80 °C. Reaction was terminated after 90 min by pouring the acid and washing it with pure water until the straw became neutral. Samples were then dried in an oven at 65 °C for 24 h.

**Figure 10:**
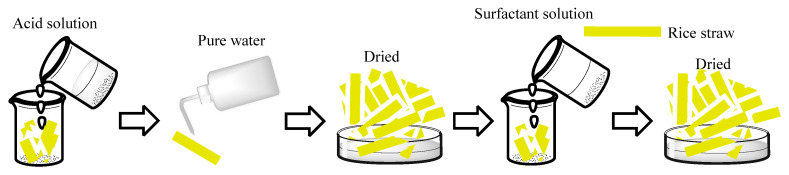
Modification of rice straw.

Then, the dried rice straw was modified with 50 mL anionic surfactant solution, and the beaker was put in the water bath at 20 °C. Samples were then dried in an oven at 65 °C for one day. The modification process of rice straw is shown in Figure 10.

### 4.3. Determination of Oil Adsorption Rate

The dried straw was placed into a homemade small iron cage of ~30 mesh, and then it was put into a 250 mL beaker with 150 mL of oil at 40 °C. They were taken out after 1.5 h to drip oil for 40 min. Finally, the straw was weighed and three readings were made on every sample to check repeatability and to obtain an average value. The oil adsorption rate was calculated as follows:

(1) $$\;Q\;=\frac{m_{2}–m_{1}}{m_{1}–m_{0}}$$

where Q is the oil absorption rate (g/g); m_0_ is the quality of the empty net (g); m_1_ is the total mass of the net and the oil-absorbing material before oil absorption (g); and m_2_ is the total mass (g) of the net and the oil-absorbing material after oil absorption. The oil absorption of the blank net was subtracted when calculating the final oil absorption.

### 4.4. Characterization of Rice Straw

In order to determine the change in the contact angle between the rice straw with pure water before and after the modification, rice straw was analyzed using contact angle measurement instrument (JC2000DS, Beijing Shangdetong Technology Co., Ltd., Shanghai, China). Infrared spectrum of rice straw, untreated and treated, was analyzed using an FT-IR spectrometer(Thermo Fisher Technology (China) Co., Ltd. Shanghai, China). All IR measurements using the KBr pellet technique (1 mg of a sample homogenized with 200 mg KBr) were performed on Fourier infrared spectrometer at room temperature ranging from 400 to 4000 cm^−1^, and each sample was scanned 32 times with a resolution of 0.4 cm^−1^, which was then recorded.

The phase composition and purity of the catalyst were analyzed by powder X-ray diffraction (JDX-3530, Shimadzu Enterprise Management (China) Co., Ltd., Beijing, China) on an XRD-6000 diffractometer with Cu Kα radiation at 40kV voltage and 15mA current. Thermogravimetric analysis (TGA) was carried out using a TGA/SDTA851 (Nanjing Huicheng Instrument Co., Ltd., Nanjing, China) instrument over the temperature range of 25–700 °C with a heating rate of 10 °C/min. The measurements were carried out in N_2_ atmosphere with a flow rate of 20 mL/min using alumina crucibles.

## Figures and Tables

**Table 1:** The physical parameters of oil from Yanchang oil field.

*μ*^30^ /(mPa·s)	Pour Point t/°C	*ρ*^20^ /(g·cm^−3^)	Resins *W*/%	Asphaltenes *W/*%	Aromatic Hydrocarbons *W*/%	Saturated Hydrocarbons *W*/%
36.9	18.5	0.886	12.1	6.8	25.2	55.9

## Data Availability

The data presented in this study are wholly available within the manuscript.
